# Analysis of treatment pathways for three chronic diseases using OMOP CDM

**DOI:** 10.1007/s10916-018-1076-5

**Published:** 2018-11-13

**Authors:** Xin Zhang, Li Wang, Shumei Miao, Hua Xu, Yuechuchu Yin, Yueshi Zhu, Zuolei Dai, Tao Shan, Shenqi Jing, Jian Wang, Xiaoliang Zhang, Zhongqiu Huang, Zhongmin Wang, Jianjun Guo, Yun Liu

**Affiliations:** 1 Department of Information, the First Affiliated Hospital, Nanjing Medical University, No.300 Guang Zhou Road, Nanjing, 210029 Jiangsu China; 2 Institute of Medical Informatics and Management, Nanjing Medical University, No.300 Guang Zhou Road, Nanjing, 210029 Jiangsu China; 3 School of Biomedical Engineering and Informatics, Nanjing Medical University, 101Longmian Avenue, Nanjing, 211166 Jiangsu China; 4 Department of Medical Informatics, Medical School, Nantong University, 19 Qixiu Road, Nantong, 226001 Jiangsu China; 5 School of Biomedical Informatics, the University of Texas Health Science Center at Houston, Houston, TX 77030 USA

**Keywords:** Treatment pathways, OHDSI, OMOP Common Data Model (CDM), Chronic diseases

## Abstract

The present study examined treatment pathways (the ordered sequence of medications that a patient is prescribed) for three chronic diseases (hypertension, type 2 diabetes, and depression), compared the pathways with recommendations from guidelines, discussed differences and standardization of medications in different medical institutions, explored population diversification and changes of clinical treatment, and provided clinical big data analysis-based data support for the development and study of drugs in China. In order to run the “Treatment Pathways in Chronic Disease” protocol in Chinese data sources,we have built a large data research and analysis platform for Chinese clinical medical data. Data sourced from the Clinical Data Repository (CDR) of the First Affiliated Hospital of Nanjing Medical University was extracted, transformed, and loaded into an observational medical outcomes partnership common data model (OMOP CDM) Ver. 5.0. Diagnosis and medication information for patients with hypertension, type 2 diabetes, and depression from 2005 to 2015 were extracted for observational research to obtain treatment pathways for the three diseases. The most common medications used to treat diabetes and hypertension were metformin and acarbose, respectively, at 28.5 and 20.9% as first-line medication. New drugs were emerging for depression; therefore, the favorite medication changed accordingly. Most patients with these three diseases had different treatment pathways from other patients with the same diseases. The proportions of monotherapy increased for the three diseases, especially in recent years. The recommendations presented in guidelines show some predominance. High-quality, effective guidelines incorporating domestic facts should be established to further guide medication and improve therapy at local hospitals. Medical institutions at all levels could improve the quality of medical services, and further standardize medications in the future. This research is the first application of the CDM model and OHDSI software in China, which were used to study, treatment pathways for three chronic diseases (hypertension, type 2 diabetes and depression), compare the pathways with recommendations from guidelines, discuss differences and standardization of medications in different medical institutions, demonstrate the urgent need for quality national guidelines, explores population diversification and changes of clinical treatment, and provide clinical big data analysis-based data support for the development and study of drugs in China.

## Introduction

Chronic diseases are the main cause of death worldwide, with an annual death toll higher than the sum of deaths caused by all other diseases. During 2011–2025, the cumulative economic losses due to non-communicable diseases (NCDs) under a “business as usual” scenario in low- and middle-income countries was estimated at US$ 7 trillion. This sum far outweighs the annual US$ 11.2 billion cost of implementing a set of high-impact interventions to reduce the NCD burden. These chronic diseases are mainly cardiovascular diseases, cancer, diabetes, and chronic respiratory diseases. The prevalence of chronic diseases not only brings great suffering to patients, but also seriously affects the development of society and the economy [1]. Therefore, studies concerning the diagnosis, treatment, and interventions for chronic disease are increasingly important.

With the continuous improvement of people’s living standards and changes of lifestyle during recent years, the aging population has increased, among whom hypertension has become a highly prevalent cardiovascular disease (CVD) [2]. According to statistical data from WHO, global fatalities caused by CVDs were 17 million in 2012, accounting for 46% of the fatalities caused by chronic diseases; and fatalities caused by hypertension and its complications were 9.4 million, making hypertension a primary risk factor influencing global disease burden [3]. The morbidity and mortality of CVDs in China are both increasing constantly. Fatalities from CVDs account for more than 40% of disease deaths in China. It was estimated that in China, there are 290 million CVD patients, 270 million of whom have hypertension [4]. There are a great variety of antihypertensive drugs, and administration of these drugs presents varies with the changes to the national essential drugs list.

Diabetes is one of the most severe and critical health issues faced by the world in the twenty-first century. The number of diabetes patient has increased progressively, year by year, and patients suffer from complications influencing their whole lives. A total of 415 million adults have diabetes, which equates to 1 in 11 adult sufferers. By 2040, this will rise to 642 million (1 adult in 10). Currently, a person dies from diabetes every 6 s (5.0 million deaths) [5, 6]. Worldwide, the medical expenses for diabetes and its complications have increased continuously at a rate of 12% per year, as diabetes induces lesions of the eye, nervous system, kidney, heart, and blood vessels and other chronic progressive lesions, which significantly affect patients’ quality of life, aggravate their economic burden, and represent a large portion of the total health expenditure [7, 8]. In 2015, the number of diabetes patients in China was 109.6 million, ranking the top in the world, and the medical expenditure related to diabetes was 51 billion USD (the second in the world). It is estimated that the number of diabetes patient in China will reach 150.7 million by 2040, and the total medical expenditure for diabetes will be 72 billion USD. Nearly half of diabetes patients (46.6%) die below the age 60 years [7, 9].

Depression is a common disease worldwide, affecting more than 300 million patients. Depression is different from average emotional fluctuation and transient emotional response to challenges in daily life. Especially, moderate and severe depression might progress into a critical disease, which significantly affects a patient and results in poor performances at work and school, and at home. For the most severe cases, depression induces suicide. Every year, more than 0.8 million people commit suicide, which is ranked the second most common cause of death in the 15–29 year-old population. It has been suggested that there might be more than 20 cases of attempted suicide for one adult death by suicide [10].

With the rapid development of information technologies, US President Obama implemented strategies of Precision Medicine in 2012, aiming to realize an organic combination of clinical data and genomics, and to discuss improvements in disease prevention and treatment precision with individualized treatment. However, there is a certain gap in relevant studies between the USA and China. With the implementation of information standardization in China, and under the context of data sharing and clinical data-related studies, the construction of platform-based database projects first solves issues concerning data standardization and sharing, and makes it possible to conduct clinical big data-based observational research. Randomized clinical trials are the foundation of medical causation evidence, which might be limited and deficient because of various factors, such as the size of the subject population, the duration of the experiment, and the scope of the target population. Based on an observational research, current treatment measures can be identified and used for comparison with new therapeutic methods; observed data can directly test clinical hypotheses and verify correct effect evaluation processes of nonrandom treatment assignment. Thus, the characters of different populations can be better understood to improve results of observational and experimental research [11, 12].

By analyzing patient diagnoses and medications, the present study compares the treatment pathways used in the First Affiliated Hospital of Nanjing Medical University, which has published normative guidelines, to explore the standardization of medication in medical institutions, provide a reference for diagnosis and treatment of chronic diseases in basic-level hospitals, and provide data support for national drug research and development via clinical big data.

## Materials and Method

### Network and Tools

Observational Medical Outcomes Partnership (OMOP) that was founded in 2008, is a public-private partnership. The research target of the partnership was gradually changed from initially protecting human health through drug safety and effectiveness monitoring to exploring effectiveness studies of clinical products with the use of observational health databases. A new collaboration was formed -- Observational Health Data Sciences and Informatics (OHDSI). This is an interdisciplinary collaboration undertaken by a multi-stakeholder group to discover the value of observational health data through large-scale analysis [13].

The Common Data Model (CDM) defines a set of uniform data standards that regulate the format and content of observational data, support observational data from different sources, and form a standardized data structure through data Extraction-Transformation-Loading (ETL). On this basis, data can be used for query and analysis. The OMOP CDM contains the 39 tables which refer to standardized vocabularies, standardized clinical data, standardized health economics, standardized health system data, standardized meta-data, and standardized derived elements.

Once a database has been converted to the OMOP CDM, evidence can be generated using standardized analytics tools. OHDSI is based on the methodological study of OMOP, and is the development and application of the method to answer real clinical problems with observational data. The researchers developed ETL tools (WhiteRabbit, Usagi et al.), data analysis tool (ACHILLES, PLATO et al.), which are used for data quality and characterization, medical product safety surveillance, comparative effectiveness, quality of care, and patient-level predictive modeling [14].

On the basis of these works, some researchers study in the network. Suchard *et al.* study in “Population-level estimation of comparative risks of Celecoxib versus non-selective NSAIDs” [15]. Wong and Schuemie *et al.* study in “Drug Utilization in Children” [16]. Huser *et al.* study in “Data Quality Study” [17]. Hripcsak *et al.* study in “Treatment Pathways in Chronic Disease” [11]. In Hripcsak’s study, they created an international data network with 11 data sources from four countries –US, UK, South Korea, and Japan. This study is the first OHDSI type of study on informatics in China. We proved the feasibility of these OHDSI type studies in China.

### Our Platform

**Fig. 1:**
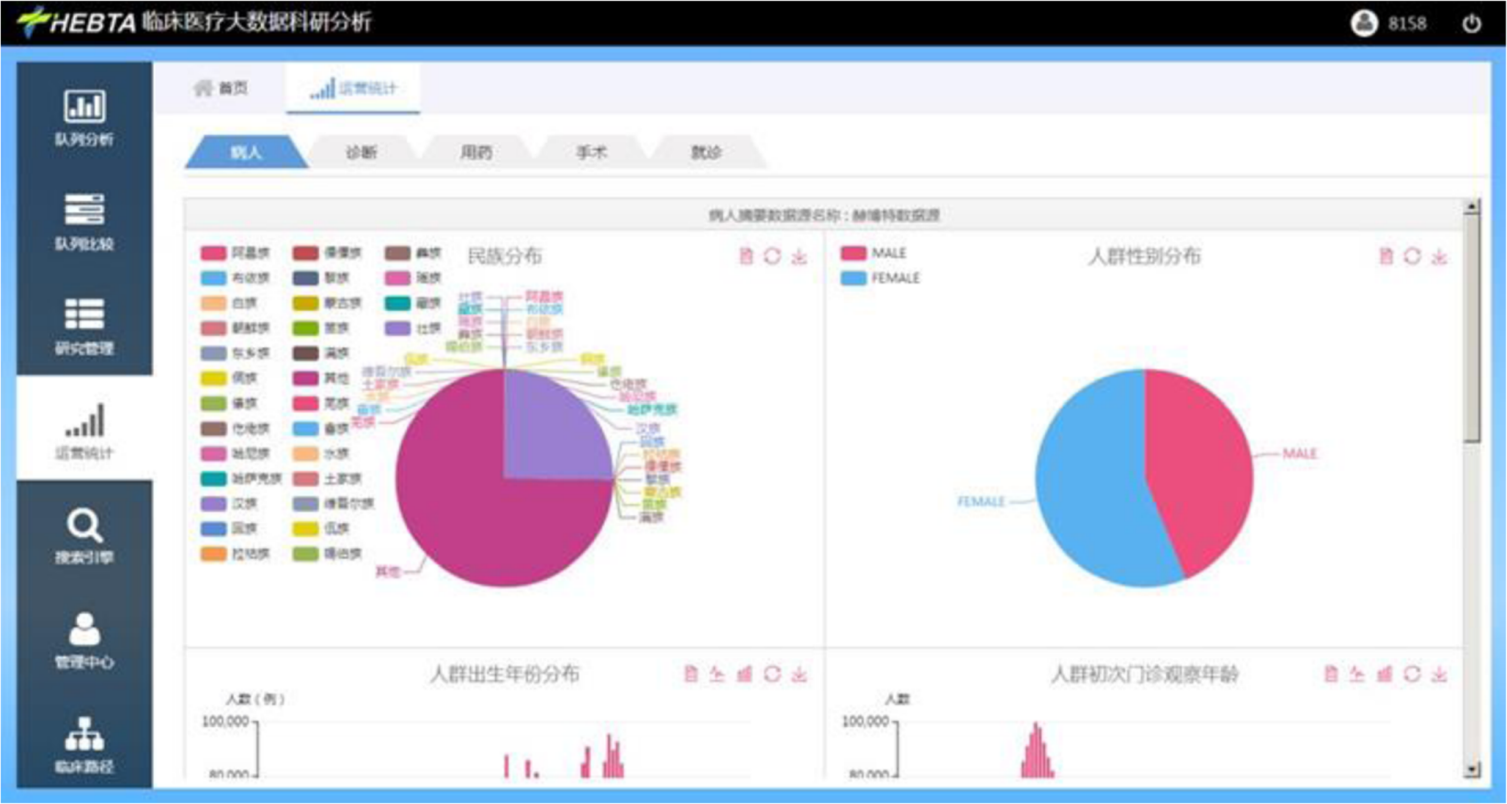
The interface of the large data research and analysis platform for clinical medical data

ALL the above mentioned OHDIS tools are used to analyze English clinical data, in order to run the “Treatment Pathways in Chronic Disease” protocol in Chinese data sources,we have built a large data research and analysis platform for Chinese clinical medical data. The platform is showed in Fig. 1. This is a set of medical data analysis and visualization display system, which provides functions such as standardization of medical data, timing analysis of medical data, specific patient cohort analysis and clinical path analysis. The platform is mainly divided into cohort analysis, cohort comparison, research management, operation statistics and search engine modules, aiming at accurate analysis of clinical medical research data. The operation statistics module makes descriptive statistical analysis of all the patients’ information in the system, which helps to understand the quality of data, and also contributes to the decision analysis of medical management.

### Data and Methods

Data concerning basic characteristics, diagnoses, and medications of 6,230,000 patients from January 1st, 2005 to December 31st, 2015 were extracted from the Clinical Data Repository (CDR) of the First Affiliated Hospital of Nanjing Medical University, and went through privacy-free and cleaning treatment to map an observational medical outcomes partnership common data model (OMOP CDM; Ver. 5.0) [13]. All patients diagnosed with hypertension, type 2 diabetes (hereinafter referred to as diabetes), or depression, and information about their medications, were increased. There were a total of 276,816 patients diagnosed with hypertension, 140,511 with diabetes, and 24,915 with depression. Patients that met the exclusion criteria, and their information, were removed, as follows.

For hypertension, the diagnosis used was hyperpiesis, and its exclusion criterion was pregnancy. For diabetes mellitus, type 2 (diabetes), the diagnosis used was diabetes and type 2 diabetes mellitus, and its exclusion criteria were pregnancy observations or type 1 diabetes mellitus. For depression, the diagnosis used was depression disorder, and its exclusion criteria were pregnancy, bipolar 1 disorder, or schizophrenia.

The index date was considered to be the first exposure to medication. The patient had to have at least 6 months of history in the database before the index date to increase the likelihood that this was a first treatment of the disease by any medication. The patient had to have at least 1 year of continuous treatment after the index date, with some medication targeted to the disease. The requirement guarantees that a patient was not transferred to another medical institution for treatment, and, therefore, the research could obtain relatively complete treatment records of the patient. A total of 34,142 patients with hypertension, 11,826 patients with type 2 diabetes, and 1519 patients with depression were enrolled in the study cohort. The year of the index date was adopted for patient grouping.

For every patient meeting the conditions of any chronic disease, drug concept IDs for the 1st round, 2nd round, 3rd round…and Nth round (N represents a certain round), were recorded until no record of a new drug for the patient were observed. Emphasis was laid on newly increased doses therapeutic drugs or the order of replaced drugs. If there was a compound drug, calculation was conducted according to contents of multiple drug ingredients.

Based on the above information, statistical analysis was conducted for every disease, and the numbers of patients treated with different treatment pathways were recorded. For example,

- Diabetes mellitus, type 2, Treatment Pathways_1, Drug_Concept_1, Drug_Concept_2, Drug_Concept_3,……, Num_Pathways_1
- Diabetes mellitus, type 2, Treatment Pathways_2, Drug_Concept_1, Drug_Concept_2, Drug_Concept_3,……, Num_Pathways_2
- Diabetes mellitus, type 2, Treatment Pathways_N1, Drug_Concept_1, Drug_Concept_2, Drug_Concept_3,……, Num_Pathways_N1
- Hypertension, Treatment Pathways_1, Drug_Concept_1, Drug_Concept_2, Drug_Concept_3,……, Num_Pathways_1
- Hypertension, Treatment Pathways_2, Drug_Concept_1, Drug_Concept_2, Drug_Concept_3,……, Num_Pathways_2
- Hypertension, Treatment Pathways_N2, Drug_Concept_1, Drug_Concept_2, Drug_Concept_3,……, Num_Pathways_N2
- Depression, Treatment Pathways_1, Drug_Concept_1, Drug_Concept_2, Drug_Concept_3,……, Num_Pathways_1
- Depression, Treatment Pathways_2, Drug_Concept_1, Drug_Concept_2, Drug_Concept_3,……, Num_Pathways_2
- Depression, Treatment Pathways_N3, Drug_Concept_1, Drug_Concept_2, Drug_Concept_3,……, Num_Pathways_N3

**Fig. 2:**
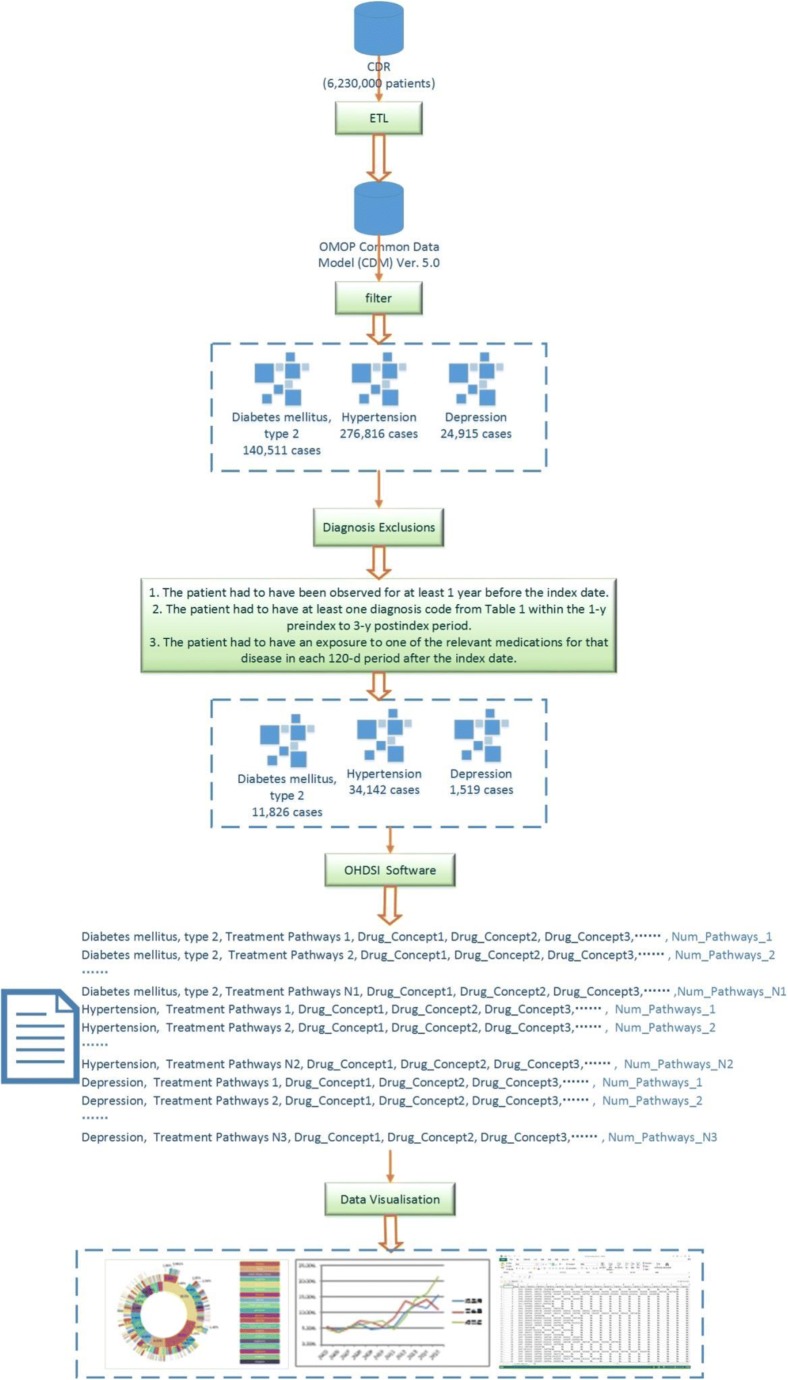
Processing Process and Data Flow. The data was extracted from CDR, then transformed, loaded to an OMOP CDM. Cases were addressed of patients with three diseases: type 2 diabetes mellitus, hypertension, and depression. The cases with exclusion diagnosis were removed. The data according to the set conditions was filtered, analyzed by some OHDSI software, and visualized to different graphs and charts

For every treatment pathway for the three chronic diseases, medications used as the 1st, 2nd, and 3rd round and the number of patients treated with the different drugs were counted. We obtained graphs and charts of different summarized results according to different factors, including diseases, time, and medications. The process and data flow are summarized in Fig. 2.

To facilitate the comparison of medication consistency among different diseases, we set three indexes: (1) The proportion of the number of patients treated with monotherapy during the whole medication sequence among the total number of patients with the disease in the cohort; (2) the proportion of the number of patients treated with common monotherapy during the whole medication sequence among the total number of patients with the diseases in the cohort; and (3) the proportion of the number of patients treated with the most common drug as the first line medication among the total number of patients with the diseases in the cohort. With these indexes, we could compare treatment pathways of different diseases in a generic way, and a higher proportion indicated relatively higher treatment consistency for the disease.

An Oracle 11 g database was used for data storage and the SQL language was used for data extraction. This is implemented with d3.js (a JavaScript library used to visualize data using web standards) for data analysis and graphics generation.

## Results

**Fig. 3:**
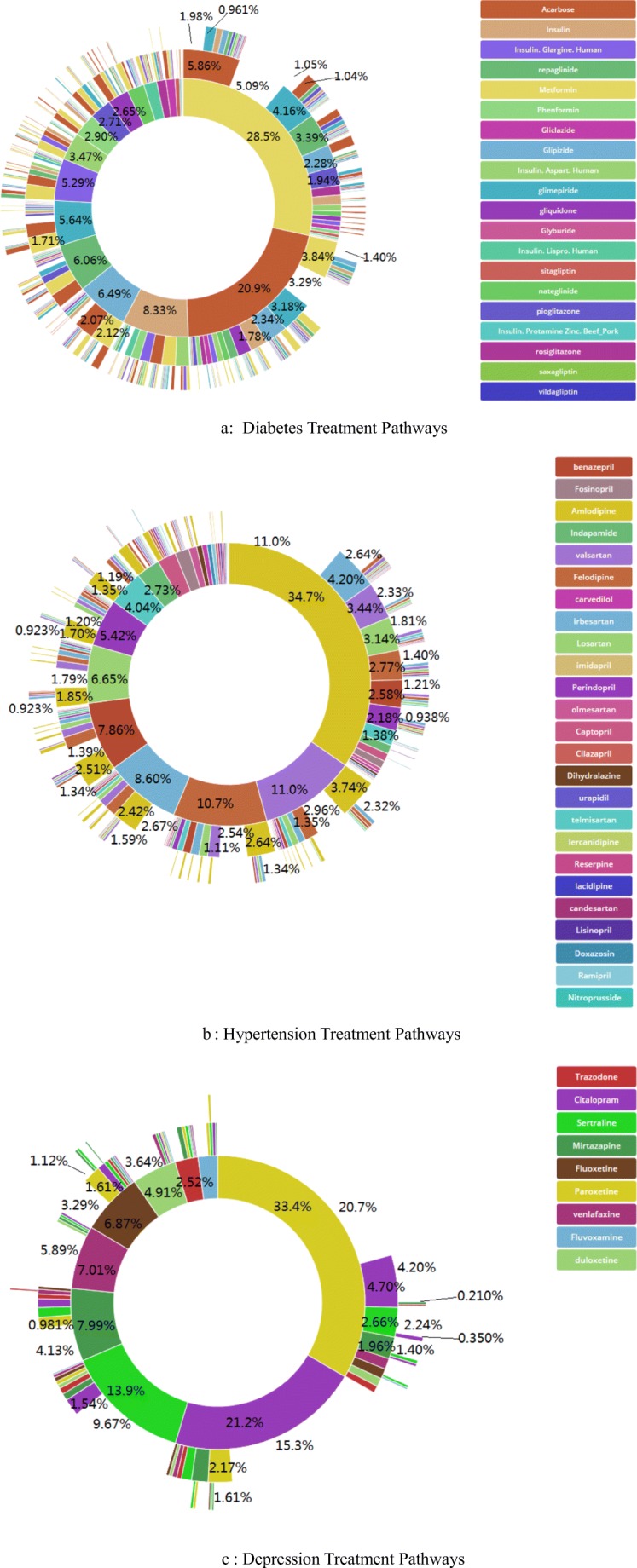
Treatment pathways for each disease, diabetes (A), hypertension (B), and depression (C). The inner circle shows the first medication related to the disease that was used by the patients, the second circle shows the second medication, and so forth. Twenty medications were recorded, but only three circles are shown in this figure

The treatment pathways for the three diseases are illustrated in Fig. 3. For diabetes, metformin was the most commonly prescribed medication; it was prescribed 28.5% of the time as the first medication and remained the only medication for 5.09% of the time. By contrast, acarbose was prescribed 20.9% of the time as the first medication and remained the only medication for 3.29% of the time. Thus, only metformin had predominance as a staring medication in our hospital. This indicted that there is certain gap between the administration of metformin in our Hospital and the first-line recommendation of *Guidelines for the prevention and treatment of type 2 diabetes mellitus in China and Standards of medical care in diabetes—2014* [18, 19] (Fig. 3A).

From the treatment pathways of hypertension shown in Fig. 3B, amlodipine was identified as the most common treatment for hypertension in our hospital; it was prescribed 34.7% of the time as the first medication and remained the only medication for 11.0% of the time. Compared with the *Guidelines for rational use of hypertension* and *Clinical Practice Guidelines for the Management of Hypertension in the Community*, this usage is consistent with the recommendation that a diuretic or calcium antagonist can be used as the drug of choice [20–22].

For the treatment pathways for depression shown in Fig. 3C, there appeared to be relatively fewer drug choices for the treatment of depression in China than those in other countries, and the treatment pathways are relatively simple. Paroxetine was administered most frequently. It was prescribed 33.4% of the time as the first medication and remained the only medication for 20.7% of the time. Citalopram was prescribed 21.2% of the time as the first medication and remained the only medication for 15.3% of the time. Sertraline was prescribed 13.9% of the time as the first medication and remained the only medication for 9.67% of the time. In the following graphs, analysis was conducted on the basis of index date to obtain data concerning replacement of new drugs and old drugs. According to the ACT system query [23], paroxetine, citalopram, and sertraline are all selective serotonin reuptake inhibitors (N06AB). These facts are consistent with the guidelines for first-line recommendation [24, 25].

It is worth noting that treatment pathways used in 85.27% of the diabetes cases, in 85.19% of the hypertension cases, and in 53.71% of the depression cases are different from other patients with the same disease in this cohort. The data suggested that within the 1.5-year observation period of the study, most patients did not use the same treatment pathways as others.

**Fig. 4:**
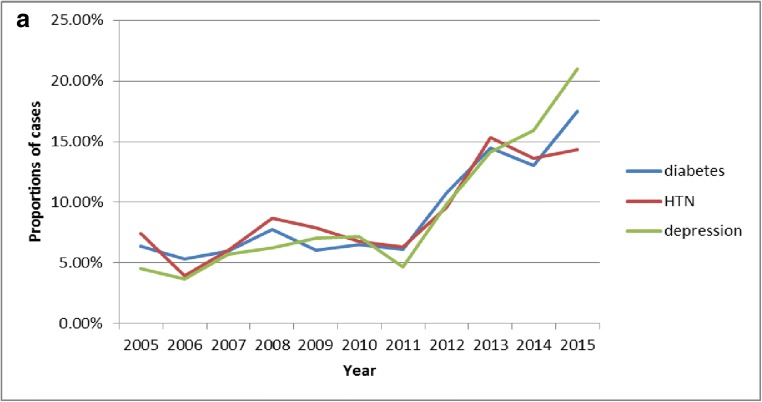

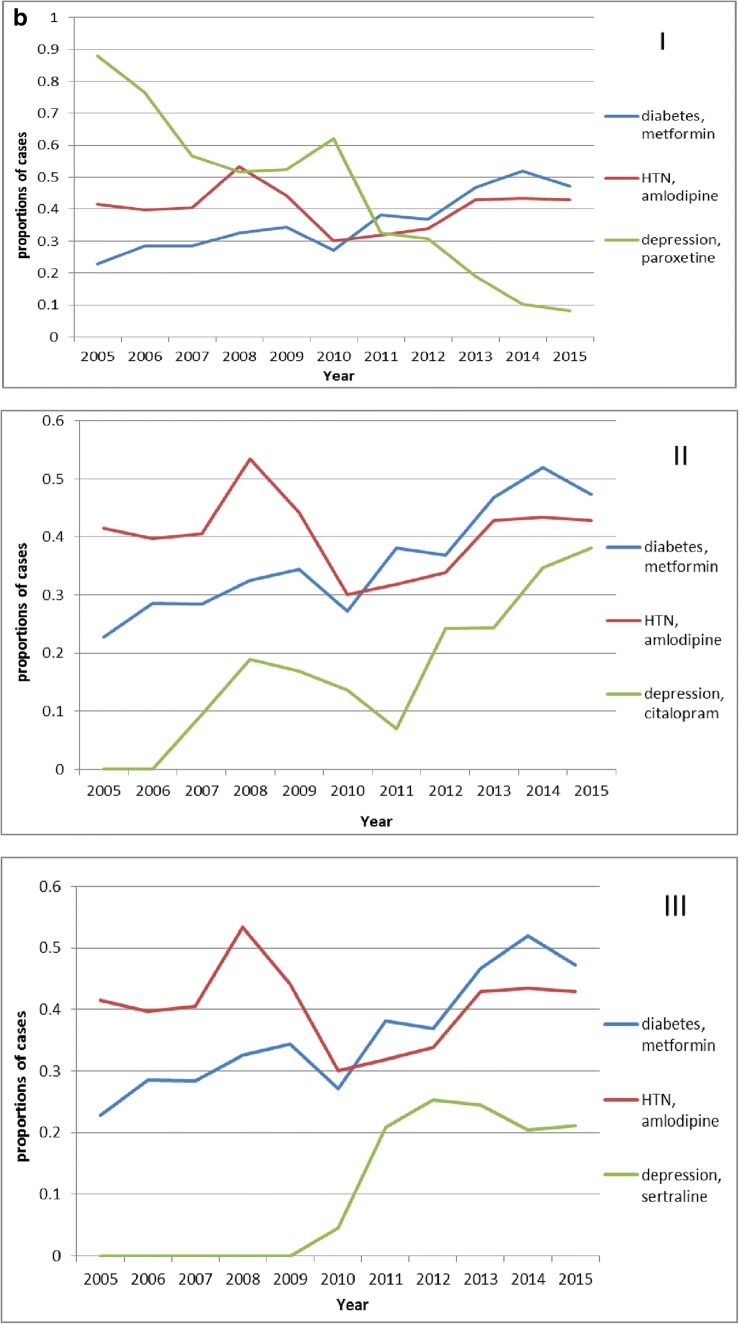

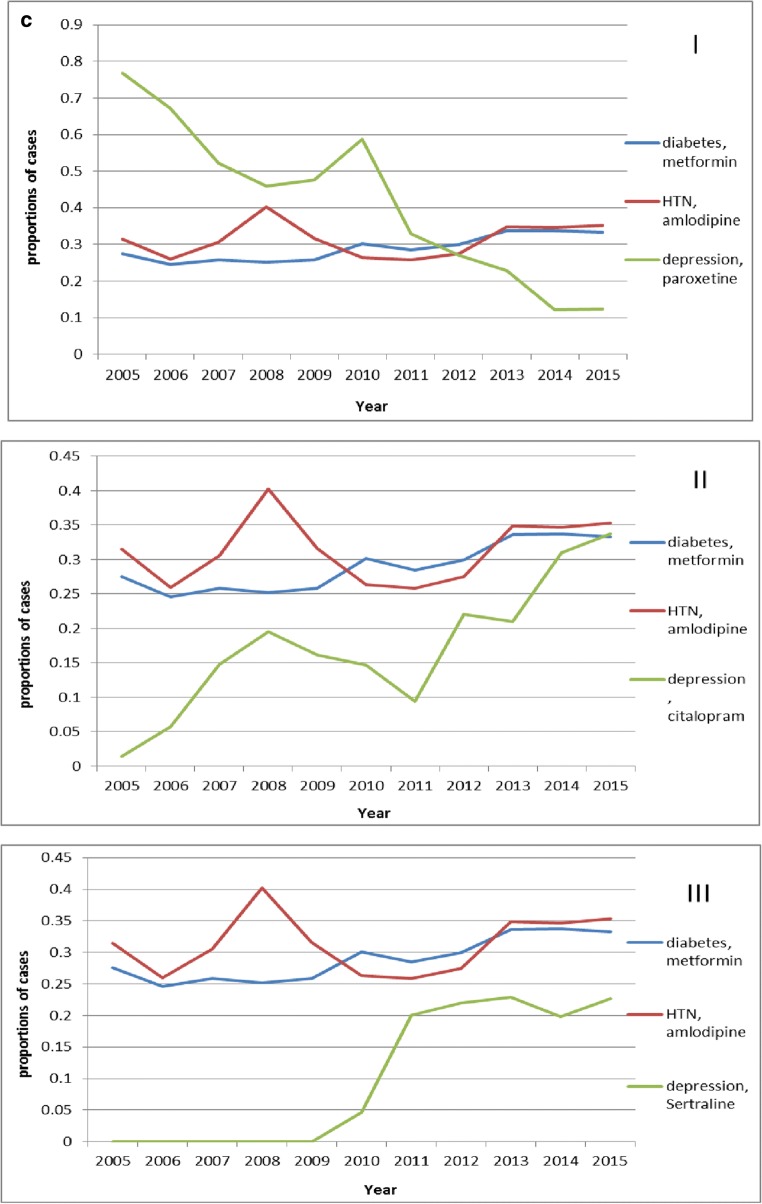
Trends for the three chronic diseases represented as three broken line graphs. A. Monotherapy proportions. The horizontal axis represents the year and the vertical axis represents the proportions of cases with only one medication in the sequence (monotherapy) for the three chronic diseases. B. The most prortions for the common monotherapies. The horizontal axis represents the year and the vertical axis represents proportions of cases in which the sequence contains only the most common monotherapy medication for that disease. In graph (I), (II), and (III), three different antidepressants are used. C. The proportions of treatment pathways begining with the most common medication. The horizontal axis represents the year and the vertical axis represents the proportions of cases in which a sequence begins with the most common starting medication for that disease. In graphs (I), (II), and (III), three different antidepressants are used

Certain trends for the three chronic diseases as are presented as three broken line graphs. Fig. 4A presents the rising trends of monotherapy for the three diseases from 2005 to 2015, and, especially after 2011, the rising trends are obvious. The percentage of patients with diabetes using monotherapy increased from 6.38% in 2005 to 17.51% in 2015. The percentage of patients with hypertension using monotherapy increased from 7.41% in 2005 to 14.35% in 2015. The percentage of patients with depression using monotherapy increased from 4.55% in 2005 to 21.00% in 2015.

Fig. 4B indicates the trends of monotherapy with the most common drugs for the three diseases. Both diabetes and hypertension cases showed increasing trends. The percentage of patients with diabetes using monotherapy with metformin increased from 22.77% in 2005 to 45.09% in 2015. The percentage of patients with hypertension using monotherapy with amlodipine increased from 41.51% in 2005 to 47.89% in 2015. For depression, the administration of paroxetine presented a declining trend, and after the release of citalopram and sertraline, the number of patients treated with monotherapy of the two drugs individually both increased gradually. The percentage of patients with depression using monotherapy with paroxetine decreased from 88.10% in 2005 to 6.15% in 2015. The percentage of patients with depression using monotherapy with citalopram was 0 in 2005, increasing to 9.43% in 2007 and then to 48.98% in 2015. The percentage of patients with depression using monotherapy with sertraline was 0 in 2005, increasing to 4.55% in 2010 and then to 16.33% in 2015.

Fig. 4C shows the trends of first round treatment with the most common medications for the three chronic diseases, and suggests that the trends are relatively stable for diabetes and hypertension, while the medication for depression varies considerably. Among the medications used for depression, paroxetine showed a declining trend, while citalopram and sertraline both showed year-on-year increasing trends. The percentage of patients with diabetes receiving first-round treatment using metformin was 27.56% in 2005 and 33.2% in 2015. The percentage of patients with hypertension receiving first-round treatment using amlodipine was 31.48% in 2005 and 35.29% in 2015. The percentage of patients with depression receiving first-round treatment using paroxetine decreased from 76.71% in 2005 to 12.30% in 2015. The percentage of patients with depression receiving first-round treatment using citalopram increased from 1.37% in 2005 to 33.73% in 2015. The percentage of patients with depression receiving first-round treatment using sertraline increased from 4.59% in 2010 to 22.62% in 2015.

## Discussion

In this study, based on privacy-free treatment and cleaning, CDR data was converted into an OMOP Common Data Model (CDM), which covered data about visits, diagnosis, prescriptions, medications, and laboratory results, and the connections among the data. With Observational Health Data Sciences and Informatics (OHDSI) tools, and collaboration with different multinational data sources in the future, we could carry out more observational research. Different data sources acquired using the same analytical procedures can highly expand the scope of data for research, which will be accelerated without the limitation of having one only data source. Meanwhile, as different analytical procedures are operated locally within the corresponding data sources, risks concerning privacy leak and data safety are avoided to allow local studies to proceed smoothly.

Both differences and similarities were revealed by comparing the treatment pathways of the three chronic diseases. The proportions of treatment with monotherapy all increased, especially in recent years, for the three chronic diseases (Fig. 4A). For diabetes, the percentage increased from 6.38% in 2005 to 17.51% in 2015. For hypertension, the percentage increased from 7.41% in 2005 to 14.35% in 2015. For depression, the percentage increased from 4.55% in 2005 to 21.00% in 2015.). With the introduction of new medications to treat depression, old medications were gradually replaced (Fig. 4B I, II, III). The percentage of patients with depression using monotherapy with paroxetine decreased from 88.10% in 2005 to 6.15% in 2015. The percentage of patients with depression using monotherapy with citalopram and sertraline increased separately from 0 in 2005 to 48.98 and 16.33% in 2015. Even with the recommendations in the guidelines, there are still not enough patients using these medications. Specifically, the proportions of metformin and amlodipine as the first-line therapy for diabetes and hypertension, respectively, are not high enough (Fig. 4C). For diabetes, the percentage was 27.56% in 2005, and 33.26% in 2015, and for hypertension, the percentage was 31.48% in 2005 and 35.29% in 2015. For the medications used to treat depression, paroxetine ranked the first in the past (76.71% in 2005), and this situation is gradually declined as the proportions occupied by newer medications, citalopram, and sertraline increased (12.30%, 33.73%, and 22.62%, respectively, in 2015). Further guidance for medication is required to improve treatment levels in basic-level hospitals, and to improve the quality of medical services in hospitals at all levels and to further standardize medication.

The overwhelming majority of the three patient types did not have the same treatment pathways as others with the same disease during the 1.5-year observation period of the study. This suggested that there is some difference between clinical practice and guideline recommendations; therefore, high-quality and effective guidelines are urgently needed, and that guideline compliance is required for treatment. Increasingly high feasibility of precision medicine and precise master of patient characters make it possible to individualize an optimal treatment pathway for every patient.

In Hripcsak’s previous study, the patient had to have at least 1 year of history in the database before the index date, and have at least 3 years of continuous treatment after the index date [11, 26]. In our study, the patient had to have at least 0.5 year of history in the database before the index date, and have at least 1 year of continuous treatment after the index date. The study design was modified due to the actual situation of our datasets. This will lead to an increase in the number of patients enrolled in the study cohort, and more study data will be available. As a consequence of this modification, there was less continuous record of patients. The accuracy of the second-line medication and the third-line medication was poorer than it was in previous study. Hripcsak mentioned that three years was chosen to ensure sufficient time to characterize a pathway, although this requirement lost patients who died within the 3-y period [11]. We chose one year so that the number of lost patients would be much smaller. We still need to consider the situation of these patients died within the 1-y period.

For further improvement, proportions of drug replacement during 1st, 2nd, and 3rd rounds can be used to partially conclude therapeutic effects of medications and to provide data basis for researches and developments of new drugs in China.

OHDSI has successfully converted health records of 1/10 global population into a common data model. With more and more institutions participating in the research cooperative program, the data scope will be expanded, and large-scale clinical big data researches will lead to study conclusions with higher universality or make contributions to understandings on population diversification and clinical treatment variations.
